# Optimization and repeatability of multipool chemical exchange saturation transfer MRI of the prostate at 3.0 T

**DOI:** 10.1002/jmri.26690

**Published:** 2019-02-15

**Authors:** Vincent Stephen Evans, Francisco Torrealdea, Marilena Rega, Mrishta Brizmohun Appayya, Arash Latifoltojar, Harbir Sidhu, Mina Kim, Aaron Kujawa, Shonit Punwani, Xavier Golay, David Atkinson

**Affiliations:** ^1^ Centre for Medical Imaging University College London London UK; ^2^ Institute of Nuclear Medicine, University College London Hospital NHS Foundation Trust, University College Hospital London UK; ^3^ Radiology Department University College London Hospital NHS Foundation Trust, University College Hospital London UK; ^4^ Institute of Neurology University College London London UK

**Keywords:** CEST, prostate, repeatability, optimization, cancer

## Abstract

**Background:**

Chemical exchange saturation transfer (CEST) can potentially support cancer imaging with metabolically derived information. Multiparametric prostate MRI has improved diagnosis but may benefit from additional information to reduce the need for biopsies.

**Purpose:**

To optimize an acquisition and postprocessing protocol for 3.0 T multipool CEST analysis of prostate data and evaluate the repeatability of the technique.

**Study Type:**

Prospective.

**Subjects:**

Five healthy volunteers (age range: 24–47 years; median age: 28 years) underwent two sessions (interval range: 7–27 days; median interval: 20 days) and two biopsy‐proven prostate cancer patients were evaluated once. Patient 1 (71 years) had a Gleason 3 + 4 transition zone (TZ) tumor and patient 2 (55 years) had a Gleason 4 + 3 peripheral zone (PZ) tumor.

**Field Strength:**

3.0 T. Sequences run: T_2_‐weighted turbo‐spin‐echo (TSE); diffusion‐weighted imaging; CEST; WASABI (for B_0_ determination).

**Assessment:**

Saturation, readout, and fit‐model parameters were optimized to maximize in vivo amide and nuclear Overhauser effect (NOE) signals. Repeatability (intrasession and intersession) was evaluated in healthy volunteers. Subsequently, preliminary evaluation of signal differences was made in patients. Regions of interest were drawn by two post‐FRCR board‐certified readers, both with over 5 years of experience in multiparametric prostate MRI.

**Statistical Tests:**

Repeatability was assessed using Bland–Altman analysis, coefficient of variation (CV), and 95% limits of agreement (LOA). Statistical significance of CEST contrast was calculated using a nonparametric Mann–Whitney *U*‐test.

**Results:**

The optimized saturation scheme was found to be 60 sinc‐Gaussian pulses with 40 msec pulse duration, at 50% duty‐cycle with continuous‐wave pulse equivalent B1 power (B1_CWPE_) of 0.92 μT. The magnetization transfer (MT) contribution to the fit‐model was centered at –1.27 ppm. Intersession coefficients of variation (CVs) of the amide, NOE, and magnetization transfer (MT) and asymmetric magnetization transfer ratio (MTR_asym_) signals of 25%, 23%, 18%, and 200%, respectively, were observed. Fit‐metric and MTR_asym_ CVs agreed between readers to within 4 and 10 percentage points, respectively.

**Data Conclusion:**

Signal differences of 0.03–0.10 (17–43%) detectable depending upon pool, with MT the most repeatable (signal difference of 17–22% detectable).

**Level of Evidence**: 2

**Technical Efficacy**: Stage 2

J. Magn. Reson. Imaging 2019;50:1238–1250.

CHEMICAL EXCHANGE SATURATION TRANSFER (CEST) imaging allows for the detection of in vivo metabolites via the continual exchange of labile protons with water. The technique has been applied in cancer imaging at clinical field strengths across a range of anatomical regions including brain,^1^, ^2^ breast,^3^, ^4^, ^5^ head and neck,^6^, ^7^ and prostate^8^, ^9^ with sequences performed in clinically feasible scan times.

A key diagnostic tool in prostate cancer detection and treatment pipeline is the use of multiparametric magnetic resonance imaging (mp‐MRI) protocols and radiologist scoring schemas, which are becoming increasingly harmonized across sites as radiological consensus continues to emerge.^10^, ^11^

While mp‐MRI has been shown to improve detection of clinically significant prostate cancer^12^ approximately a third of cases remain equivocal.^13^, ^14^ In these cases, there would be a clear clinical benefit if additional information could be provided by imaging to support diagnosis and reduce the need for biopsies.

CEST imaging is sensitive to changes in the concentrations and the pH environment of exchangeable protons found in metabolites such as lactate, citrate, and other mobile proteins and peptides that exhibit altered behavior as part of metabolomic changes that occur in prostate cancer.^15^ Jia et al^8^ demonstrated that amide proton transfer ratio (APTR) measurements in regions of prostate cancer were significantly higher than in benign peripheral zone (PZ) regions, while Takayama et al^9^ analyzed CEST data from 66 prostate tumors that suggested that amide proton transfer (APT) measurements of Gleason 7 lesions are higher than for Gleason 6, 8, and 9 lesions and for noncancerous PZ tissue. Lorentzian fitting of CEST z‐spectra gives the ability to derive information from several different exchange effects that would otherwise be convolved into a single asymmetry measurement and, to the best of our knowledge, has not previously been explored in the prostate at 3.0 T.

In order for the clinical potential of the technique to be explored fully, an optimization of the acquisition and analysis, and evaluation of the repeatability of the results, is needed.

The present work has two primary objectives. The first is to optimize an acquisition and postprocessing protocol suitable for z‐spectrum fitting analysis of prostate data acquired at 3.0 T with clinically feasible scan times. The second is to evaluate the intra‐ and intersession repeatability of both the fitting and asymmetry metrics derived from the optimized protocol. It is hypothesized that the repeatability scores of the CEST fitting metrics will outperform those from asymmetry analysis.

## Materials and Methods

### *Subjects*

The recruitment of healthy volunteers and patients was approved by the local Institutional Ethics Board. Written informed consent was obtained prior to all examinations.

Five healthy male volunteers (age range 24–47 years; median age: 28 years) were recruited. Two of these volunteers were initially scanned as part of the sequence and postprocessing optimization steps described below. All five healthy volunteers then attended two separate scanning sessions (range of interscan intervals: 7–27 days; median interval: 20 days) where data for the repeatability analysis was collected.

Patients were identified by a radiologist with 10 years of experience (coauthor S.P.) during local multidisciplinary team meetings. Two patients with transrectal ultrasound (TRUS) biopsy‐proven tumors, both of whom were under active surveillance for lesions scoring 5/5 on PI‐RADS, with maximum lesion size >10 mm and whose biopsies had taken place over 4 months prior to recruitment were recruited and made a single attendance. Patient 1 (age 71) had a right‐sided Gleason 3 + 4 TZ tumor with prostate‐specific antigen (PSA) concentration of 8.4 ng/ml and patient 2 (age 55) had a right‐sided Gleason 4 + 3 PZ tumor with PSA concentration of 6.2 ng/ml. PSA measurements were made 2 months prior to the research scans.

### *MRI Protocol*

Data were acquired with a 3.0 T Achieva MRI scanner (Philips Healthcare, Best, The Netherlands) using a 32‐channel cardiac coil. All volunteer and patient scans were performed axially in the feet‐first position. A T_2_‐weighted (T_2_w) whole‐volume turbo‐spin‐echo (TSE) acquisition and, in patients, a diffusion‐weighted imaging (DWI) acquisition was performed at the start of the session. These were then used to plan the CEST scan. In patients, both of which had known and previously classified tumors, prior mp‐MRI was also available to support the radiologists when planning. In healthy volunteers, a single‐slice was positioned at the largest axial cross‐section of the prostate. In patients, the slice was positioned at the largest axial cross‐section of the tumor as determined by radiology research fellows (A.L. and H.S.) with access to prior clinical imaging. The shim voxel was aligned with the imaging plane.

The T_2_w scan was an axial multishot TSE (TSE factor = 16). Echo time (TE) = 100 msec, repetition time (TR) = 4000 msec, field‐of‐view (FOV) = 180 × 180 mm^2^, resolution = 224 × 218 with 30, 3‐mm thick slices. Refocusing control and fat suppression were off. A parallel imaging acceleration (SENSE) factor of 1.3 (RL) was used. The number of signal averages (NSA) was 1. Scan duration was 3 minutes 27 seconds.

The DWI scan used an axial multislice Cartesian single‐shot echo planar imaging (EPI) readout. TE = 79 msec, TR = 2360 msec, FOV = 220 × 220 mm^2^, resolution = 168 × 168 with 14, 5‐mm slices. NSA = 6. Images were acquired using a single b‐factor of 2000. Spectral presaturation with inversion recovery (SPIR) fat suppression was applied. Scan duration was 2 minutes 10 seconds.

The CEST scan readout was an axial single‐shot TSE readout. TE = 14 msec, TR = 5100 msec, FOV = 140 × 140 mm^2^, resolution = 64 × 63 with a single slice thickness 4 mm. Refocusing control was set to 120° and SPIR fat suppression was applied. No parallel imaging acceleration was used. NSA = 1. The scan duration was 5 minutes 42 seconds.

### *Offset Sampling Frequencies*

Saturated images were acquired with 0.25 ppm spacing between ±5 ppm, with additional measurements at ±5.5, ±6.0, ±6.5, ±7.0, ±7.5, ±10.0, ±15.0, ±20.0, ±25.0, ±30.0, ±100.0, and ± 300.0 ppm allowing for sampling of the broad semisolid MT contribution. An unsaturated reference image was acquired for z‐spectrum normalization.

### *WASABI*

B_0_ maps were acquired using a WASABI (water shift and B_1_) sequence that was adapted from parameters outlined in the literature.^16^ Data were acquired at 20 saturation frequency offsets, evenly spaced between ±3 ppm. A 5‐msec block saturation pulse of flip angle 284° (B_1CWPE_ = 3.7 μT) was applied before each readout. The readout parameters and slice‐planning were the same as for the CEST scan. The duration of each WASABI scan was 41 seconds.

### *Data Analysis*

Data processing was performed using in‐house developed software written using MatLab (MathWorks, Natick, MA, R2016a).

B_0_ inhomogeneity corrections were carried out by interpolating the CEST z‐spectra to 1 Hz (0.008 ppm) frequency intervals and shifting the spectra on a voxel‐by‐voxel basis using B_0_ maps generated using WASABI data.

#### *Lorentzian Fitting*

The direct saturation (DS), CEST, and nuclear Overhauser effect (NOE)^17^, ^18^, ^19^ pools were modeled using Lorentzian lineshapes,^20^, ^21^, ^22^ described as a function of the saturation frequency, ω by:

(1) $$L_{i}\left(\omega ,\omega_{0i},\Gamma_{i},A_{i}\right)=A_{i}\frac{1}{2\pi }\frac{\Gamma_{i}}{{\left(\omega -\omega_{0i}\right)}^{2}+{\left(0.5\Gamma_{i}\right)}^{2}}$$

Where ω_o*i*_ is the offset frequency of pool *i*, Γ*i* is the full‐width‐half‐maximum (FWHM), and *A*_*i*_ is a scaling factor.

The CEST effects were jointly modeled using a single Lorentzian to mitigate the risks of overfitting. This Lorentzian was centered at the APT frequency offset of 3.5 ppm and is henceforth referred to as the amide pool. Similarly, a single Lorentzian, located at –3.5 ppm, was used to account for the NOE effect.^22^, ^23^

The MT contribution to the z‐spectrum was modeled using a super‐Lorentzian curve,^21^, ^24^, ^25^ which is described by:

(2) $$\mathrm{SL}\left(\omega ,\omega_{0\mathrm{SL}},T_{2}^{b},A_{\mathrm{SL}}\right)=A_{\mathrm{SL}}\int_{0}^{\frac{\pi }{2}}\text{dθsinθ}\sqrt{\frac{2}{\pi }}\frac{T_{2}^{b}}{\left|3\cos^{2}\theta -1\right|}e^{-2{\left(\frac{\left(\omega -\omega_{0\mathrm{SL}}\right)T_{2}^{b}}{\left|3\cos^{2}\theta -1\right|}\right)}^{2}}$$

where $T_{2}^{b}$ is the T_2_ of the MT pool, ω_0*SL*_ is the central offset frequency of the lineshape relative to water, and *A*_*SL*_ is a scaling factor.

The offset frequency of the MT contribution to the z‐spectrum has been seen to vary across tissues.^23^, ^26^, ^27^ This issue is discussed by van Zijl et al^28^ and Hua et al^26^ and is likely due to a combination of causes including differences in the constituents of the macromolecular pool across tissues, which is thought to contain both slow‐ and fast‐exchanging contributors. The MT offset frequency was estimated by fitting the z‐spectra from many voxels using only data points outside of the ±15 ppm range, where CEST and NOE contributions are negligible. The median offset from this analysis was used for all subsequent fits and the super‐Lorentzian lineshape was symmetrically interpolated between ±20 ppm using a cubic‐spline to remove the pole that occurs close to its central frequency.

A whole‐fit frequency offset term, *h*, was included in the fit equation to account for imperfect B_0_‐field inhomogeneity corrections and a vertical constant signal term *v* was also included to account for the effects of noise during z‐spectrum normalization. After fitting, both the normalized data points and the amplitude parameters extracted from the fit were renormalized using the fitted vertical offset value (using a voxelwise multiplication by 1/(1‐v)). This ensured that the data and fit results were properly normalized.

The general overall fit equation for a renormalized z‐spectrum with contributions from *n* pools (including DS, CEST, and NOE effects) and an MT pool is given by:

(3) $$M_{z}\left(\omega \right)=1-\sum_{i=1}^{n}L_{i}\left(\omega -h,\omega_{0i},\Gamma_{i},A_{i}\right)-\mathrm{SL}\left(\omega -h,\omega_{0\mathrm{SL}},T_{2}^{b},A_{\mathrm{SL}}\right)$$

Fitting analyses were performed using a nonlinear least squares algorithm and the fit expression described in Eq. 3 customized for a four‐pool model (DS, amide, NOE, MT), with all data points weighted equally. The primary output metrics of interest from the fitting were taken to be the heights of the amide, NOE, and MT peaks.

#### *Asymmetry Analysis*

The asymmetric magnetization transfer ratio (MTR_asym_) at offset frequency ω is calculated by the expression ${\mathrm{MTR}}_{\text{asym}}\left(\omega \right)=\frac{M_{z}\left(-\omega \right)-M_{z}\left(\omega \right)}{\omega_{0}}$. For this analysis, the MTR_asym_ was calculated at ω = 3.5 ppm as per previous prostate APT work.^8^, ^9^

### *Saturation Scheme and TR Optimization*

A pulsed‐saturation scheme was used, comprised of a train of sinc‐Gaussian shaped pulses with parameters: pulse duration (τ_p_), interpulse‐delay (τ_d_), saturation flip angle (θ), and the number of pulses (*N*). Z‐spectra were acquired in a healthy volunteer using a range of values of *N* (20 to 60) to determine the number of pulses required to achieve saturation steady‐state.

The saturation flip angle was subsequently optimized by acquiring data from a second healthy volunteer using a range of values of saturation flip angle θ and selecting the flip angle that maximized the signal from the fitted amide and NOE pools obtained using the four‐pool model described by equation 3.

A range of TR values between 2 and 6 seconds were explored by acquiring unsaturated images repeatedly in a healthy volunteer and comparing the total absolute signal observed in each case. These results were cross‐referenced with values quoted in the literature for the T_1_ of the prostate at 3.0 T^29^ and a selection was made to allow for >95% signal recovery between TSE readouts.

### *Region of Interest Analysis*

Regions of interest (ROIs) were drawn in a single‐session by coauthor H.S., with 10 years of experience in prostate radiology, on unsaturated CEST reference scans from all healthy volunteer and patient scans. This was done with a graphical user interface (GUI) that was created using an in‐house developed MatLab script. For healthy volunteers, ROIs were drawn in the right and left PZ, right and left TZ, and right and left obturator internus muscles. In patients, the same ROIs were drawn, however in the affected zone (TZ or PZ) one ROI was drawn in the tumor and one in apparently‐uninvolved‐tissue (AUT), ie, not necessarily contralaterally.

Readers had access to prior clinical mp‐MRI reports and pathology results and the T_2_w and DWI images acquired during this study. This was to ensure that the tumor was localized in the acquired images as accurately as possible by both readers to allow for subsequent evaluation of the signal differences between different regions.

A second reader (coauthor M.B.A.) with 5 years of experience in prostate radiology independently redrew the ROIs. Repeatability and tumor contrast analyses were performed using both sets of ROIs.

### *Repeatability in Healthy Volunteers*

The B_0_‐corrected z‐spectra from all voxels within each ROI were averaged to generate a single z‐spectrum per ROI that was fitted using the four‐pool model to extract amide, NOE, and MT measurements, and the MTR_asym_ was calculated. Bland–Altman plots were used to evaluate the intrasession and intersession repeatability of the technique.^30^ For intrasession repeatability, the results from the first scan of each session were compared with the results from the second scan of the same session, with scans being separated by a total of 6 minutes 23 seconds. For intersession repeatability, the results from the first scan of the first session were compared with the results of the first scan of the second session, and similarly with the second scan of each session. The coefficient of variation (CV) was calculated in the standard way for each metric (amide, NOE, MT, and MTR_asym_). The 95% limits of agreement (LOA), derived in each case from the Bland–Altman analysis, are the values within which 95% of the differences between measurements are expected to lie, and the bias is the mean difference between scans. In the ideal case of perfect repeatability with no noise, the LOA, bias, and CV are all zero.

### *Contrast in Tumor Tissue*

Maps of the amide, NOE, and MT peak‐heights, and the MTR_asym_ were generated by performing voxel‐wise analysis of the patient scans. Boxplots of the signal from all voxels within tumor and AUT ROIs were created. The significance of the observed image contrast between tumor and AUT in patient scans was evaluated by treating each group of voxels within an image as an independent sample and applying a nonparametric Mann–Whitney *U*‐test with *P* < 0.05 taken to be significant.

## Results

### *Acquisition and Fitting Protocol Optimization*

**Figure 1:**
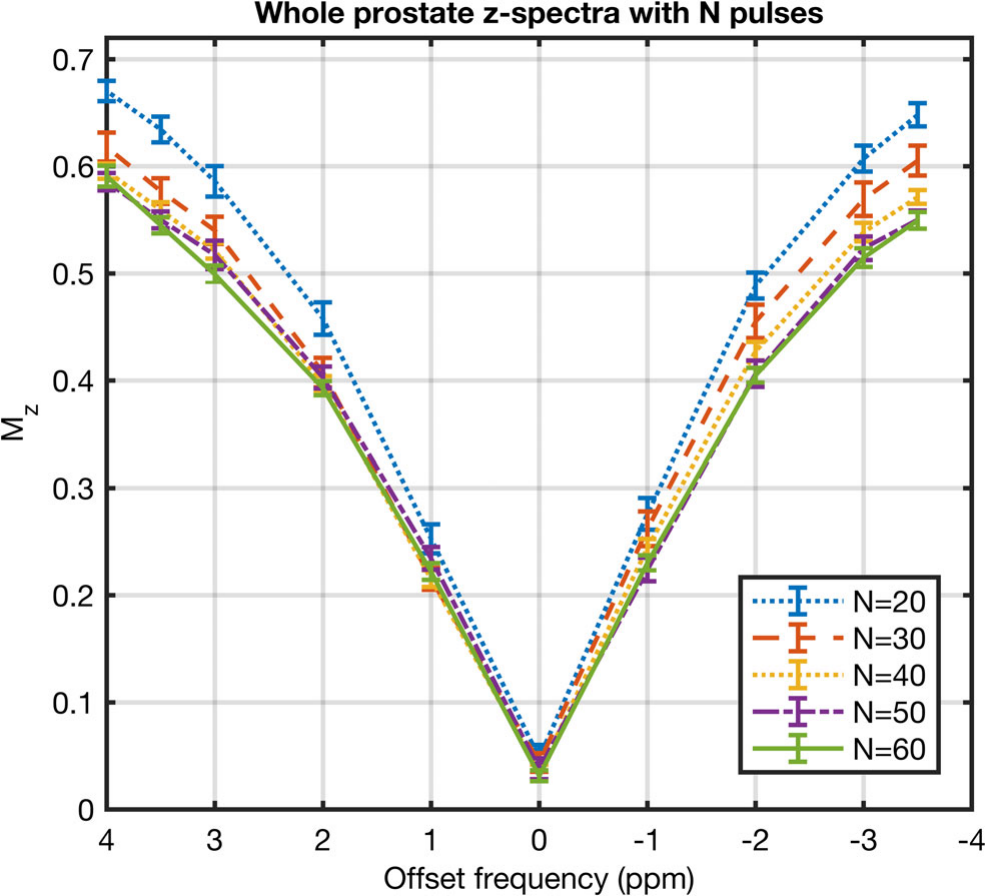
Z‐spectra from large ROIs drawn over the whole prostate when using saturation trains with *N* = 20, 30, 40, 50, and 60 Gaussian pulses. The error bars show the standard deviation of the signal across all voxels within each ROI. The z‐spectrum approaches saturation steady‐state with increasing *N*.

Figure 1 shows the z‐spectra from an ROI drawn over the whole prostate for different numbers of saturation pulses, *N*. Saturation steady‐state is approached with increasing *N* and differences between subsequent z‐spectra became smaller with each increment of 10 pulses. The trade‐off between small gains in saturation and increased scan time led to the selection of 60 pulses for the final saturation scheme. For the given saturation timings (τ_p_ = τ_d_ = 40 msec) this corresponds to 4.8 seconds of total saturation time.

In keeping with reference values from the literature^29^ and data that were acquired in a healthy volunteer (data not shown), a TR of 5 sec or above was found to allow for >95% signal recovery of M_z_ between readouts. The final TR was chosen to be 5.1 sec to accommodate the TSE readout (245 msec) and 4.8 sec of saturation.

**Figure 2:**
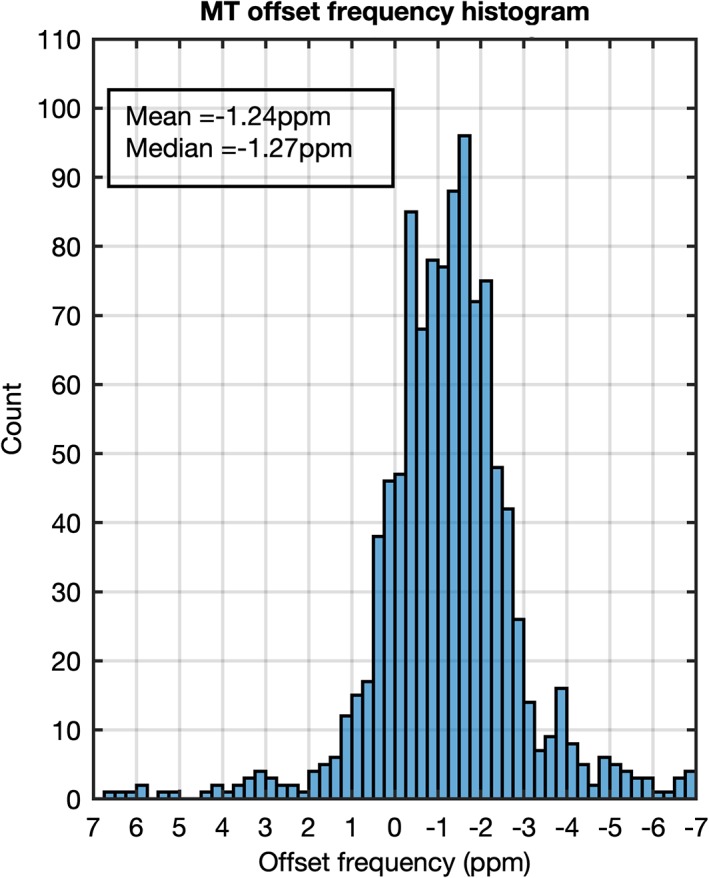
Histogram showing the distribution of offset frequencies of the fitted MT super‐Lorentzian across prostate voxels taken from five healthy volunteers when fitting only data points outside of the ±15 ppm range.

Figure 2 shows the histogram distribution of super‐Lorentzian offset frequencies when fitting only MT data points outside of the ±15 ppm range. The median offset frequency was –1.27 ppm with 25^th^ and 75^th^ percentiles at –2.04 ppm and –0.04 ppm, respectively. Based on these data, the offset of the super‐Lorentzian was fixed at –1.27 ppm for all subsequent analysis. This is similar to values used in other Lorentzian fitting work.^23^

**Figure 3:**
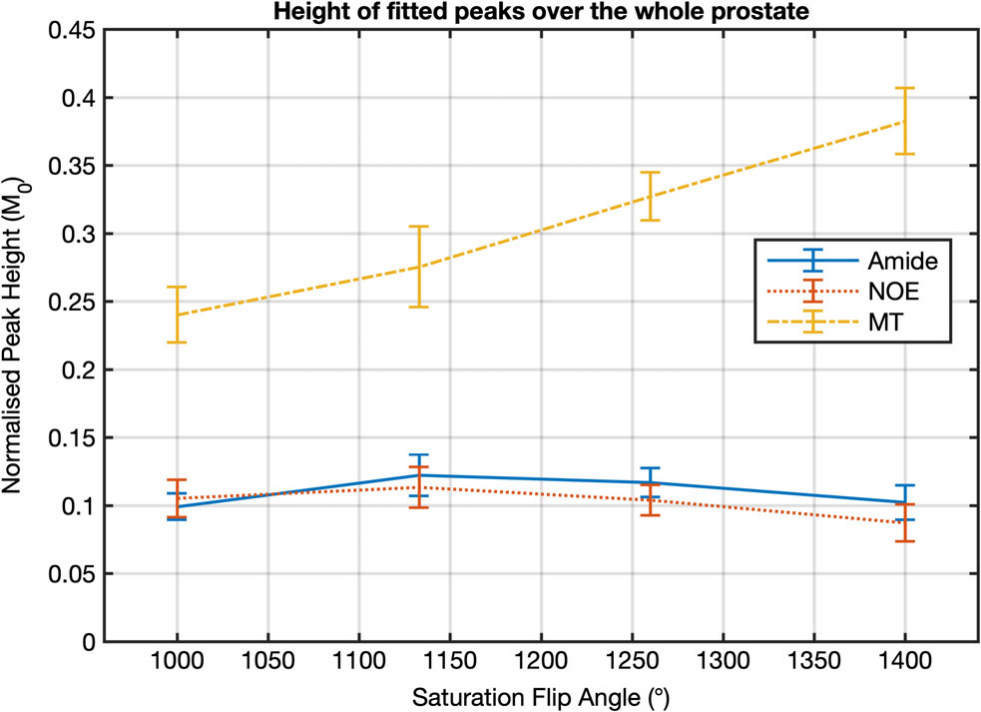
A plot of the mean heights of the fitted amide, NOE, and MT peaks over all voxels of a whole prostate for a range of saturation flip angles. Error bars show the standard deviation over all voxels. The amide and NOE signals were both maximized using a flip angle of 1133°.

The heights of the fitted amide, NOE, and MT peaks as a function of saturation flip angle are shown in Fig. 3. The MT signal increased with increasing saturation power and the mean signals from the amide and NOE pools across the whole prostate were jointly maximized with a saturation flip angle of 1133°, corresponding to a B_1CWPE_ of 0.92 μT. The fit parameters used in the final analysis are summarized in Table 1.

**Table 1:** Starting Values and Upper and Lower Bounds for All Parameters Used by the Z‐spectrum Fitting Algorithm

Z‐spectrum contributor	Lineshape	Parameter	Starting value	Lower bound	Upper bound
Water	Lorentzian	*ω*_0_ (ppm)	0.0000	–0.0001	0.0001
		Γ (ppm)	1.0	0.5	12.0
		*A* (A.U)	0.8	0.1	1.0
Amide	Lorentzian	*ω*_0_ (ppm)	3.500	3.499	3.501
		Γ (ppm)	2.0	0.5	12.0
		*A* (A.U)	0.1	0.0	1.0
NOE	Lorentzian	*ω*_0_ (ppm)	–3.500	–3.501	–3.499
		Γ (ppm)	2.0	0.5	12.0
		*A* (A.U)	0.1	0.0	1.0
MT	Super‐Lorentzian	*ω*_0*SL*_ (ppm)	–1.27000	–1.27001	–1.26999
		$T_{2}^{b}$ (μs)	10	1	50
		*A*_*SL*_ (A.U)	0.500	0.001	1.000
Horizontal Offset (whole‐fit)	Constant	*h* (ppm)	0.0	–0.3	0.3
Vertical Offset (whole‐fit)	Constant	*v* (A.U)	0.0	–0.1	0.1

### *Repeatability in Healthy Volunteers*

**Figure 4:**
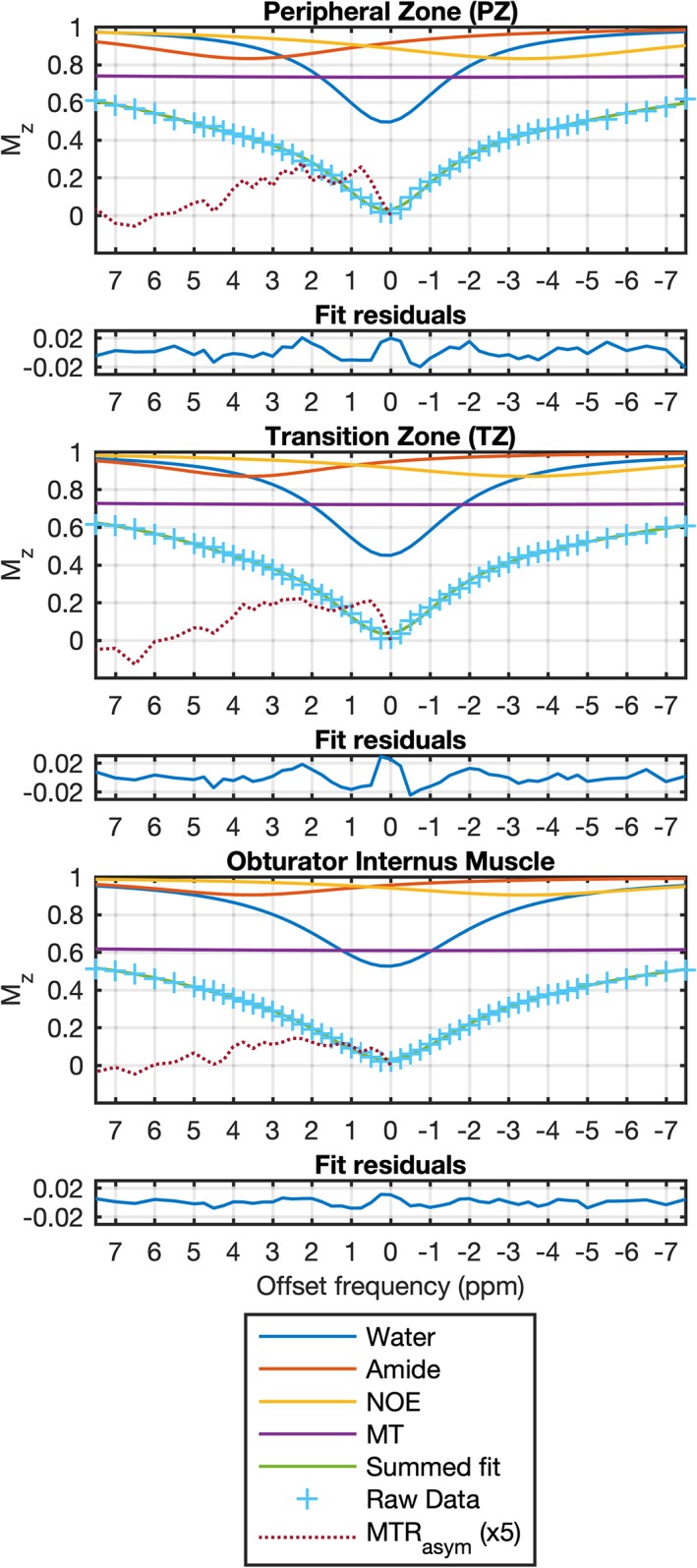
Representative normalized z‐spectra, fit results, and fit residuals from ROIs drawn in the PZ, TZ, and muscle in a single healthy volunteer, showing only the ±7 ppm range. The four‐pool fit results include contributions from water, amide, NOE, and MT. The MTR_asym_ has been scaled ×5 for improved visibility. The residual differences between the raw data and the fits are all less than 0.028.

Representative fitted z‐spectra from ROIs drawn in the PZ, TZ, and obturator internus muscle of a single healthy volunteer are shown in Fig. 4.

**Figure 5:**
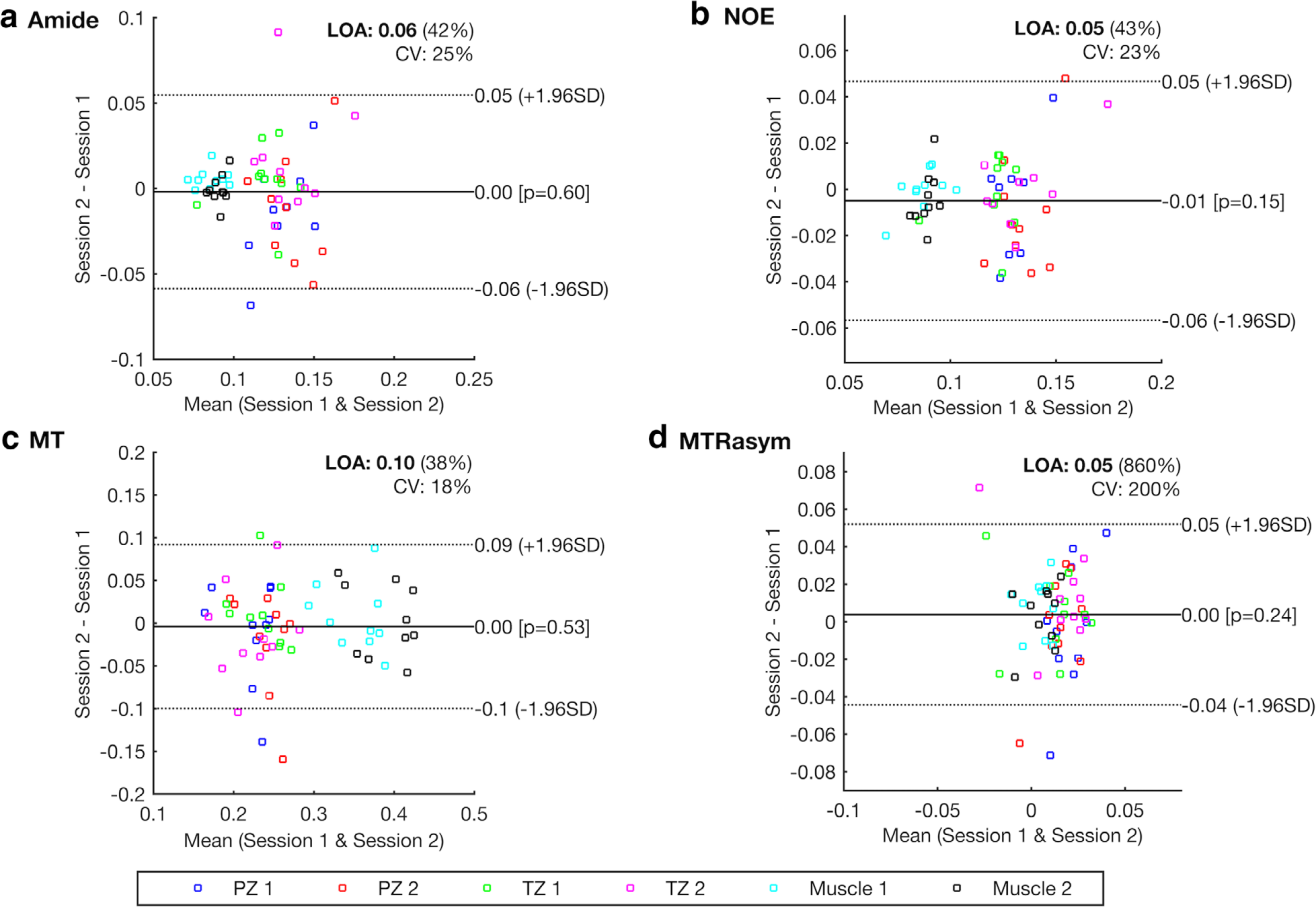
Bland–Altman plots showing the intersession repeatability of the heights of the fitted **(a)** amide, **(b)** NOE, and **(c)** MT peaks, and **(d)** the MTR_asym_ measurements, using ROIs drawn by reader 1. Plots include the 95% LOA expressed in absolute terms and as a percentage, the bias, and the CV.

Figure 5a–d show Bland–Altman plots for the intersession repeatability of the heights of the fitted amide, NOE, and MT lineshapes and the MTR_asym_ measurements, respectively, using the ROIs drawn by reader 1 (intrasession plots are not shown). The plots include the CV and the LOA expressed in units of *M*_0_, which equates to *M*_*z*_(∞) = 1 for a normalized z‐spectrum.

The intrasession CVs of the amide, NOE, MT, and MTR_asym_ measurements in healthy volunteers using ROIs drawn by reader 1 were found to be 20%, 19%, 9.5%, and 150%, respectively. The corresponding intersession CVs were found to be 25%, 23%, 18%, and 200%, respectively.

The full CV, LOA, and bias values for amide, NOE, MT, and MTR_asym_ measurements made using ROIs drawn by both readers are shown in Table 2 along with the intraclass correlation (ICC) coefficients between readers.

**Table 2:** Coefficients Of Variation (CV), 95% Limits of Agreement (LOA) and Bias of the Intrasession and Intersession Bland–Altman Plots Generated for the Amide, Nuclear Overhauser Effect (NOE), Magnetization Transfer (MT), and Asymmetric Magnetization Transfer Ratio (MTR_asym_) Signals in Five Healthy Volunteers

	Intrasession					
	CV		LOA		Bias	
CEST Metric	Reader 1	Reader 2	Reader 1	Reader 2	Reader 1	Reader 2
Amide	20%	20%	0.05	0.05	0.00	0.00
NOE	19%	18%	0.04	0.04	0.00	0.00
MT	9.5%	7.4%	0.05	0.04	0.00	0.00
MTR_asym_	150%	140%	0.04	0.03	0.00	0.00
ICC	0.997		0.539		—	

	Intersession					
	Reader 1	Reader 2	Reader 1	Reader 2	Reader 1	Reader 2
Amide	25%	22%	0.06	0.05	0.00	–0.01
NOE	23%	19%	0.05	0.04	–0.01	0.00
MT	18%	15%	0.10	0.08	0.00	0.00
MTR_asym_	200%	200%	0.05	0.05	0.00	0.00
ICC	0.999		0.835		—	

Results are shown for analysis done using regions of interest drawn by two separate readers. The intraclass correlation (ICC) coefficients are also provided.

The LOA gives an indication of the threshold for reliable detectability of signal differences. Therefore, based on these data, we would expect to be able to reliably detect differences in the amplitudes of the fitted peaks from repeated scans within a single session of 0.04–0.05 (33–38% for amide and NOE and 17–22% for MT) or higher. The bias is close to zero in every case.

### *Signal Differences in Patients*

**Figure 6:**
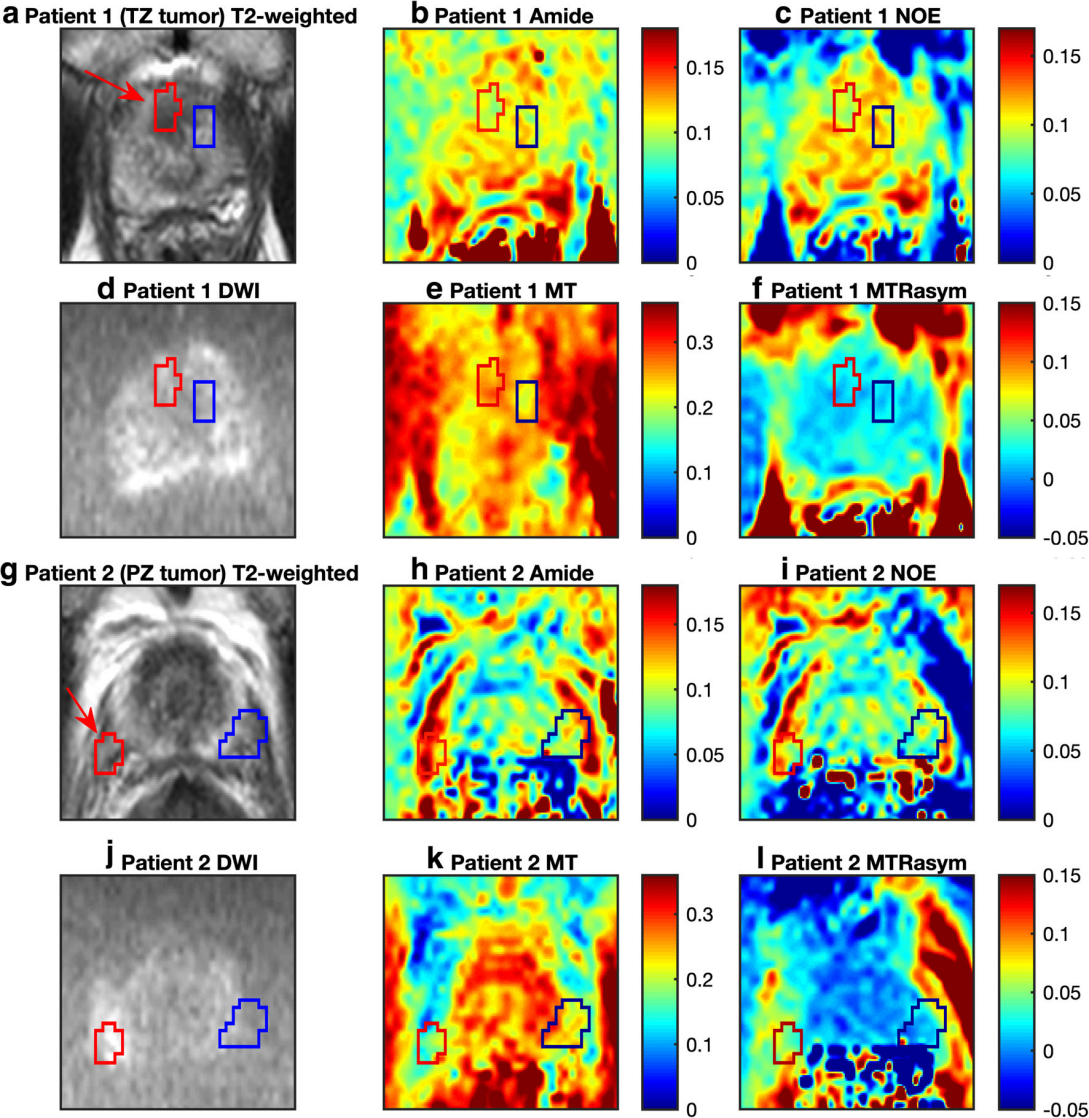
Voxelwise maps of the heights of the fitted amide, NOE and MT peaks, and the MTR_asym_ measurements for **(a–f)** patient 1 with a TZ tumor and **(g–l)** patient 2 with a PZ tumor. The tumor and AUT ROIs are highlighted in red and blue, respectively, and the tumors are highlighted with a red arrow on the T_2_w images. Visually, slight hypointensities are observed in the amide and NOE signal in the region of TZ tumor with corresponding hyperintensity in the MT signal and no significant change in the MTR_asym_. Conversely, hyperintensities in the amide and NOE signals and hypointensities in both the MT and MTR_asym_ signals are observed in the region of PZ tumor, although the hyperintense amide and NOE signals are not localized only to the region of tumor.

Maps of the amide, NOE, MT, and MTR_asym_ signal intensities from each patient are shown in Fig. 6 alongside the corresponding T_2_w and DWI (b = 2000) images. DWI is particularly useful for identifying regions of PZ tumor as seen in the figure. Tumor and AUT ROIs are overlaid onto the images, with tumor marked with a red arrow on the T_2_w images.

**Figure 7:**
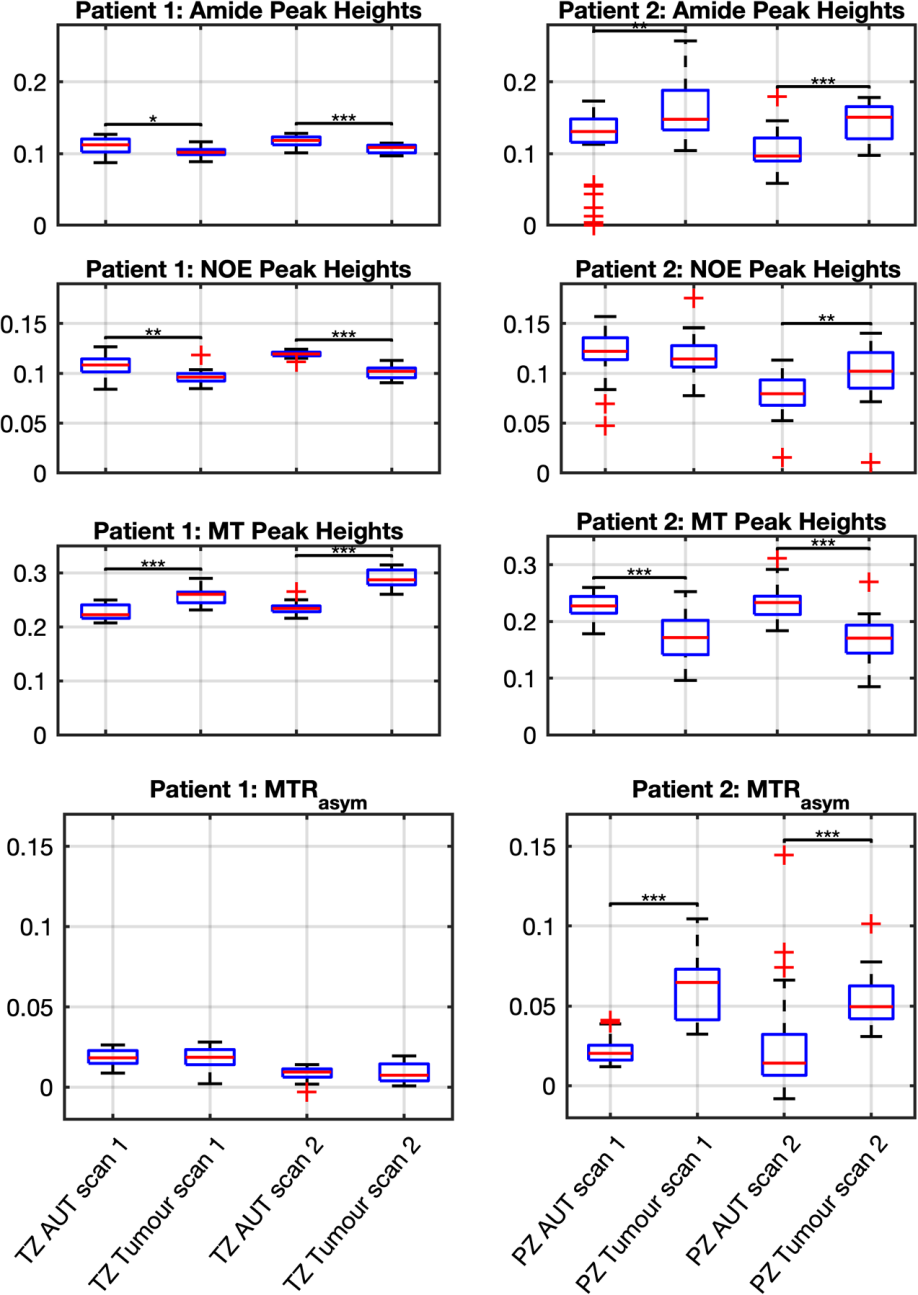
Boxplots showing the fitted amide, NOE, and MT peak heights and MTR_asym_ measurements from all voxels within tumor and AUT ROIs drawn by reader 1 for **(a)** both scans of patient 1 with a TZ tumor, **(b)** both scans of patient 2 with a PZ tumor. The red line indicates the median value of all voxels within the ROI, with the bottom and top edges of the blue box indicating the 25^th^ and 75^th^ percentiles, respectively. The whiskers extend to the most extreme data points not considered outliers. Data points that were more than 1.5× the interquartile range away from the top or bottom of the box were classed as outliers and are plotted individually using red crosses. Significant signal differences between tumor and AUT within a given scan (as calculated using the Mann–Whitney *U*‐test) are denoted using single, double, or triple asterisks representing *P* < 0.05, *P* < 0.01, and *P* < 0.001, respectively.

Boxplots of the amide, NOE, and MT signal, and the MTR_asym_ values from all voxels in AUT and tumor ROIs are shown in Fig. 7 for both patients and both readers. Statistical significance of signal differences between regions were assessed using a nonparametric Mann–Whitney *U*‐test with significance levels of *P* < 0.05, *P* < 0.01, and *P* < 0.001 denoted in the boxplots by *, **, and ***, respectively.

The median amide, NOE, MT, and MTR_asym_ signals in regions of tumor and AUT, the difference in median signals between these regions (Δ), and the corresponding significance values, *P*, are fully tabulated in Table 3. Measurements showing significant differences between tumor and AUT are highlighted in bold.

**Table 3:** Median amide, NOE, MT, and MTR_asym_ Signal Measurements in TZ AUT and TZ Tumor (Patient 1) and PZ AUT and PZ Tumor (Patient 2)

Patient 1 (TZ tumor)								
	Scan 1							
	Reader 1				Reader 2			
Median signal	TZ AUT	TZ Tumor	Δ	*P*	TZ AUT	TZ Tumor	Δ	*P*
Amide	**0.113**	**0.102**	**–0.011 (–9.7%)**	**0.020**	**0.116**	**0.104**	**–0.012 (–10.3%)**	**0.042**
NOE	**0.109**	**0.096**	**–0.012 (–11.0%)**	**0.001**	**0.116**	**0.098**	**–0.018 (–15.5%)**	**0.016**
MT	**0.222**	**0.260**	**0.038 (17.1%)**	**<0.001**	0.247	0.254	0.007 (2.8%)	0.357
MTR_asym_	0.018	0.019	<0.001 (0.0%)	0.928	**0.014**	**0.023**	**0.009 (64.3%)**	**0.017**

	Scan 2							
	Reader 1				Reader 2			
Median signal	TZ AUT	TZ Tumor	Δ	*P*	TZ AUT	TZ Tumor	Δ	*P*
Amide	**0.119**	**0.109**	**–0.010 (–8.4%)**	**<0.001**	0.112	0.112	<0.001 (0.0%)	0.668
NOE	**0.120**	**0.102**	**–0.017 (–14.2%)**	**<0.001**	0.114	0.104	–0.010 (–8.8%)	0.065
MT	**0.234**	**0.287**	**0.053 (22.7%)**	**<0.001**	0.261	0.275	0.014 (5.4%)	0.342
MTR_asym_	0.009	0.007	–0.002 (–22.2%)	0.884	**0.007**	**0.015**	**0.008 (114.3%)**	**0.029**

Patient 2 (PZ tumor)								
	Scan 1							
	Reader 1				Reader 2			
Median signal	PZ AUT	PZ Tumor	Δ	*P*	PZ AUT	PZ Tumor	Δ	*P*
Amide	**0.131**	**0.148**	**0.017 (13.0%)**	**0.010**	0.142	0.140	–0.002 (–1.4%)	0.456
NOE	0.122	0.115	–0.008 (–6.6%)	0.334	0.122	0.123	<0.001 (0.00%)	0.898
MT	**0.227**	**0.172**	**–0.056 (–24.7%)**	**<0.001**	**0.242**	**0.203**	**–0.039 (–16.1%)**	**<0.001**
MTR_asym_	**0.020**	**0.065**	**0.045 (225%)**	**<0.001**	**0.018**	**0.028**	**0.010 (55.6%)**	**0.042**

	Scan 2							
	Reader 1				Reader 2			
Median signal	PZ AUT	PZ Tumor	Δ	*P*	PZ AUT	PZ Tumor	Δ	*P*
Amide	**0.097**	**0.151**	**0.054 (55.7%)**	**<0.001**	**0.091**	**0.127**	**0.036 (39.6%)**	**<0.001**
NOE	**0.080**	**0.102**	**0.023 (28.8%)**	**0.004**	**0.075**	**0.100**	**0.025 (33.3%)**	**<0.001**
MT	**0.233**	**0.171**	**–0.063 (–27.0%)**	**<0.001**	**0.273**	**0.188**	**–0.085 (–31.1%)**	**<0.001**
MTR_asym_	**0.014**	**0.050**	**0.035 (250%)**	**<0.001**	**0.009**	**0.038**	**0.030 (333.3%)**	**<0.001**

Data are shown for both scans of both patients using ROIs drawn by both readers. The signal difference, Δ, is expressed both as an absolute value and also as a percentage of the AUT signal. All associated *P*‐values were calculated using the Mann–Whitney *U*‐test and are included. Significant differences in signal between tumor and AUT are highlighted in bold. Differences in repeated signal difference measurements (Δ) between scans and readers are broadly in line with the LOA values calculated earlier. The most consistent signal differences are found in the MT pool, where signal in tumor often varies by over 0.04, thereby exceeding the best‐case intrasession LOA for MT. The second scan of patient 2 shows the largest overall signal differences and consistency between readers where both the amide and MT signal changes in PZ tumor are greater than the LOA for these two pools.

Visually, hypointensities of both the amide and NOE signals are observed in the region of TZ tumor when compared with apparently uninvolved TZ (patient 1), with corresponding hyperintensity of the MT signal.

For patient 2, the opposite contrast effect is observed, with hyperintensity of amide and NOE signals within the region of PZ tumor relative to apparently uninvolved PZ and a corresponding relative hypointensity of the MT signal. However, it should be noted in this case that the hyperintensities of amide and NOE signals within the tumor ROI appear to be part of broader structural features that are not localized to the tumor itself.

In the five volunteers, MT was the most repeatable parameter (see Table 2) with the lowest CV values and a threshold for signal detection of 0.04–0.05 (17–22%) or higher. In the PZ tumor patient, MT showed significant amplitude decreases for both scans and for both readers with signal changes in the range –0.039 to –0.085 (16–31%), consistent with the repeatability threshold.

## Discussion

In the current work, we optimized an acquisition and postprocessing pipeline for multipool CEST imaging of the prostate at 3.0 T and evaluated the repeatability of the technique in healthy volunteers. We also subsequently applied it to two patients with proven prostate cancer.

CEST sequence parameters including number of pulses, saturation powers, and the TR were tuned to maximize the heights of the fitted CEST and NOE peaks and the offset frequency of the fitted MT peak was also estimated.

The combined scan time of the CEST and WASABI scans was clinically acceptable at 6 minutes 23 seconds. The pulse duration and interpulse delay parameters were not explored during the optimization, as prior experimentation on this scanner had indicated that saturation trains of longer than ~1 sec were not possible when using duty cycles above 50% due to specific absorption rate (SAR) and hardware restrictions. A pulse duration of 40 msec was chosen because it gives rise to spectral selectivity approximating the frequency sampling that was used (0.25 ppm or 32 Hz in the central region of the z‐spectrum).

As expected, the intrasession repeatability scores were found to be superior to the intersession repeatability scores in every case, as indicated by lower CV and LOA values.

Bland–Altman analysis showed that the CV values for all fit results (both intrasession and intersession) were 25% or lower. The intrasession 95% LOA for amide, NOE, and MT amplitude differences were found to be between 0.04–0.05 (17–38%). The corresponding intersession values for amide and NOE were between 0.04–0.06 (35–43%) and for MT this rose to 0.08–0.10 (33–38%). Therefore, the threshold for detectable differences in fitted peak amplitudes in all cases ranged from 0.04–0.10 (17–43% depending on pool). While inspection of the LOA values between readers suggests reasonable interreader agreement, the ICC calculated for the intrasession variability between readers was particularly low, in part due to a low number of observations over a small range.

By comparison, a recent study of the repeatability of apparent diffusion coefficient (ADC) measurements in prostate mp‐MRI at 3.0 T found limits of agreement ranging from 13.91% to 60.49% using an endorectal coil.^31^ This puts the repeatability of CEST fitting metrics within the repeatability range of established ADC scans from the mp‐MRI protocol.

The fitted MT peak was found to be the most repeatable measurement as quantified by CV and showed the most consistent change across both scans and readers in the PZ tumor patient.

This is perhaps not surprising, as it is the broadest contribution to the z‐spectrum and all other contributions sit nested within it in the ±5 ppm range. Magnetization transfer imaging (MTI) traditionally involves acquiring unsaturated images and images saturated at an offset typically >1 kHz off‐resonance. Recent work by Arlinghaus et al^32^ showed good repeatability of quantitative MT (qMT) imaging of the breast at 3.0 T and Barrett et al^33^ demonstrated that MTI shows promise for prostate cancer detection at 3.0 T.

It was considered whether the fitting model was able to distinguish decreases in T_2_ (causing broadening of the water peak) from increases in MT signal. If the distinction could not be made, we would expect to see an inverse correlation between the T_2_w signal and MT height across the prostate voxels. No such correlation was observed (data not shown), which allayed concerns that the model was unable to distinguish between these two effects. This is attributed to the fact that MT points acquired between ±5.5 and ± 300 ppm allowed for proper characterization of the broad MT resonance.

The interreader differences in CV measurements for the fitted pools (amide, NOE, MT) are all smaller than four percentage points, with ICC of >0.99 suggesting that interreader variability is not the main source of variation.

As hypothesized, there is a marked difference between the levels of repeatability found using the fitting method, which showed maximum intrasession and intersession CV values of 20% and 25%, respectively, and the repeatability of the MTR_asym_ measurements, which showed maximum intrasession CV of 150% and an intersession CV of 200%.

The CV values for MTR_asym_ repeatability are poorer than for the fitting metrics. The LOA values for MTR_asym_, while larger than those reported in other endogenous CEST repeatability studies,^4^, ^6^ were similar to or slightly lower than those of the fitted amide and NOE peaks. Both of these values may be improved by using a sequence optimized for MTR_asym_ imaging, but that lies outside the scope of this work, which is primarily focused on z‐spectrum fitting.

All MTR_asym_ calculations in this work were made using the single‐offset approach described in previous prostate APT work.^8^, ^9^ As an alternative, the use of multiple offset frequency points in the MTR_asym_ calculation may reduce the effects of noise, and therefore improve the repeatability. For illustration, the CV values were recalculated using the mean MTR_asym_ values over three offset frequencies (3.25, 3.5, and 3.75 ppm) and the intrasession CV values from both readers were reduced to 110%, with the intersession CV values dropping to 170% and 160%. These changes are attributed to noise averaging, although the optimal choice of integration range and its physiological significance would require further optimization and consideration.

Our results suggest that, when using the acquisition protocol outlined in this work, the fit‐model described produces more repeatable CEST measurements than MTR_asym_ analysis. This is attributed to a reduction in the influence of noise when using whole z‐spectrum fitting methods.

In the patient data the signal differences were most pronounced and showed the best agreement between readers for the second scan of the PZ tumor patient. In this case the amide, NOE, and MTR_asym_ signals all showed significant increases in the region of tumor, and the MT signal was significantly reduced. While not all of these signal differences were larger than the previously measured LOAs for respective pools, the statistical significance was evaluated between groups of voxels drawn from within individual images and therefore relate to image contrast from a single subject and timepoint, not absolute signal measurements across multiple subjects and multiple timepoints. The results of the statistical significance tests should be interpreted in these terms.

The MTR_asym_ measurements are lower than those reported by Takayama et al,^9^ who observed MTR_asym_ values in the approximate range of 0.5–6% in PZ tissue and 3–8% in Gleason 7 tissue using a higher saturation power of 2.0 μT. However, the MTR_asym_ signal enhancement in the PZ tumor (found to be ~3% of M_0_, averaged over readers and scans), was broadly consistent in magnitude with the signal changes observed by Takayama et al.^9^ The magnitude of the significant signal differences between AUT and both tumor types were found to be in the range 0.01–0.06, which is consistent with the magnitude of changes expected based on previous studies.^8^, ^9^

In terms of diagnostic prostate imaging, a comparison can be drawn between CEST and magnetic resonance spectroscopy (MRS), which are sources of alternative but complementary metabolic information. CEST provides chemically‐ and pH‐weighted information derived from exchangeable protons across many metabolites, while MRS provides metabolite‐specific profiling,^6^ often focusing on the ratio of choline to citrate.^6^ While MRS has already been discussed in the context of mp‐MRI,^10^ CEST is an imaging technique capable of providing higher spatial resolution than MRS in comparable scan times (the CEST voxel size for this work is 2.2 mm^2^ compared with 6.9 mm^2^ 34 and 5.9 mm^26^ in two studies on prostate MRS). MRS is well‐suited to the characterization of known lesions or tumors under active surveillance, but the higher in‐plane resolution of CEST is an advantage when screening for unconfirmed lesions. CEST has already shown promise in cancer imaging,^4^, ^6^, ^35^ and further investigation will allow for exploration of its potential utility.

There are several limitations of the study. No antispasmodic drug was given as part of this research and bowel motion may affect the results. Motion correction of CEST data can be difficult due to changes in image contrast between saturation frequencies; however, methods currently being developed to address this could be applied in future work.^36^

To prevent overfitting, the number of free parameters cannot be too high. For this reason, in this work we used a four‐pool model with a single CEST pool and a single NOE pool. Optimization of the offset frequencies of these two pools was attempted by fitting many voxels with relaxed constraints on the upper and lower bounds of the offset frequencies, but the analysis did not converge (data not shown). We believe this to be due to the use of a reduced number of pools, the broadness of CEST resonances at 3.0 T, and the fact that different subregions of the prostate may have different CEST pool offsets. In response to this, reference values were used for the CEST and NOE offset frequencies.^22^, ^23^ In particular the amide resonant frequency of 3.5 ppm was selected because at the low B1 saturation powers we used, the saturation efficiency for the slow‐exchanging amides is higher than for amine and hydroxyl groups. Future CEST studies at field strengths above 3.0 T may better characterize z‐spectrum features in the prostate that will inform future fitting models. Generalization of these models across sites will require the consolidation of in‐house postprocessing scripts after sufficient technical development and validation has taken place.

The horizontal offset term varied smoothly across the images and values were largely contained within the –0.02 ppm to 0.04 ppm range (data not shown), suggesting that fitting artifacts in the prostate due to poor B_0_ correction are not a major concern when performing z‐spectrum fitting with a horizontal offset. For this reason, regularization of the horizontal offset constant (for example, using IDEAL‐fitting as per Zhou et al^37^) was not applied, although this could be applied in future work. Variation of the vertical offset parameter across the prostate (with values largely confined to within a range of ±0.01, data not shown) was not smoothly varying across the field of view, but as this term is included to correct for noise, this was expected.

A single‐slice readout was chosen for the benefit of having a shorter readout time and the slice thickness was 4 mm. Partial volume effects may have influenced the results, although the slice positioning was centered at the largest cross‐section of the gland in healthy volunteers, and across the largest cross‐section of the tumors (which were both >10 mm in diameter) in patients. It is expected that this will have helped to minimize partial volume effects in this study. As a clinical tool, the protocol would benefit from an increased number of slices with thickness <4 mm to provide greater coverage and minimize these effects.

Optimization of saturation parameters for maximal absolute amide and NOE signals was performed in healthy volunteers using data from the whole prostate, including PZ and TZ subregions. The chosen saturation power of 0.92 μT was broadly consistent with saturation powers used in other endogenous CEST studies utilizing z‐spectrum fitting that utilize B1_CWPE_ in the region of 1.0 μT.^23^, ^37^ For optimal cancer detection, a protocol needs to provide an adequate level of signal and contrast between AUT and tumor and variations in T_1_ and T_2_ values, pH and metabolite concentrations between regions may influence the optimal parameter set. Further work using patients may refine the protocol for cancer detection.

Multipool CEST imaging protocols similar to the one outlined in this work may be able to integrate traditional MTI metrics with semiquantitative CEST measurements to provide more detailed exchange‐based parametric information in support of mp‐MRI protocols for the imaging of prostate cancer.

The accuracy of repeatability scores and evaluation of CEST contrast in prostate tumors would be improved with the inclusion of more subjects, but the numbers included in this study provide a benchmark for repeatability and CEST contrast in tumors similar to previous works^6^, ^9^ and these initial results may be used to power future prostate CEST studies.

In summary, we optimized a full‐z‐spectrum acquisition and fitting protocol suitable for prostate imaging on a 3.0 T scanner within clinically feasible scan times. The repeatability of the fitting metrics are comparable to other mp‐MRI scans and are seen to be more repeatable than MTR_asym_. This demonstration of z‐spectrum fitting at 3.0 T and quantification of the repeatability of in vivo CEST metrics on a clinical scanner^4^, ^6^, ^35^ is a necessary step towards the translation of CEST techniques to the clinic.

A study with a larger patient cohort is required to draw any conclusions about the magnitude of signal changes in disease and the work presented here may inform both the acquisition protocol and the powering of such a study.

Matlab scripts used in this analysis are available at: https://github.com/vsevans/CEST.
